# Internal validity of the Swedish Maternal Health Care Register

**DOI:** 10.1186/1472-6963-14-364

**Published:** 2014-08-30

**Authors:** Kerstin Petersson, Margareta Persson, Marie Lindkvist, Margareta Hammarström, Carin Nilses, Ingrid Haglund, Yvonne Skogsdal, Ingrid Mogren

**Affiliations:** Department of Clinical Sciences, Obstetrics and Gynecology, Umeå University, Umeå, Sweden; School of Health and Social Studies, Dalarna University, Falun, Sweden; Umeå School of Business and Economics, Department of Statistics, Umeå University, Umeå, Sweden; Department of Clinical Science and Education, Södersjukhuset, Karolinska Institutet, Stockholm, Sweden; Department of Research and Development, Västernorrland County Council, Västernorrland, Sweden; Primary Health Care, Parental and Child Health Care, Östersund, Sweden; Primary Health Care, Maternal Health Care Unit, Örebro, Sweden

**Keywords:** Validity, Degree of coverage, National quality register, Medical records, Pregnancy outcomes, Antenatal care

## Abstract

**Background:**

The Swedish Maternal Health Care Register (MHCR) is a national quality register that has been collecting pregnancy, delivery, and postpartum data since 1999. A substantial revision of the MHCR resulted in a Web-based version of the register in 2010. Although MHCR provides data for health care services and research, the validity of the MHCR data has not been evaluated. This study investigated degree of coverage and internal validity of specific variables in the MHCR and identified possible systematic errors.

**Methods:**

This cross-sectional observational study compared pregnancy and delivery data in medical records with corresponding data in the MHCR. The medical record was considered the gold standard. The medical records from nine Swedish hospitals were selected for data extraction. This study compared data from 878 women registered in both medical records and in the MHCR. To evaluate the quality of the initial data extraction, a second data extraction of 150 medical records was performed. Statistical analyses were performed for degree of coverage, agreement and correlation of data, and sensitivity and specificity.

**Results:**

Degree of coverage of specified variables in the MHCR varied from 90.0% to 100%. Identical information in both medical records and the MHCR ranged from 71.4% to 99.7%. For more than half of the investigated variables, 95% or more of the information was identical. Sensitivity and specificity were analysed for binary variables. Probable systematic errors were identified for two variables.

**Conclusions:**

When comparing data from medical records and data registered in the MHCR, most variables in the MHCR demonstrated good to very good degree of coverage, agreement, and internal validity. Hence, data from the MHCR may be regarded as reliable for research as well as for evaluating, planning, and decision-making with respect to Swedish maternal health care services.

## Background

### Health data registers and quality registers

Nordic countries have a long tradition of using population-based health data registers to monitor the general population. These health data registers include the Swedish Cause of Death Cause Register (1952), the Swedish Cancer Register (1958), the Norwegian Medical Birth Register (1967), and the Swedish Medical Birth Register (1973) [1]. Swedish health data registers are regulated by the Health Data Law in the Swedish code of statues (1998:543) and it is compulsory for patients, as well as for the health care services, to participate in these registers [2]. The health data registers use standardized data collection procedures, enabling surveillance of the health status of the population [3]. In addition, these registers are available to researchers [3–6]. Over the last several decades, a growing number of national quality registers surveying specific medical areas have been established in Sweden. Quality registers have been initiated and are administered by professional associations from different medical areas. In contrast to health data registers, participation in quality registers is voluntary for both patients and health care providers. That is, patients can choose not to contribute their individual data to a quality register. Quality registers are regulated by the Swedish code of statutes (2008:355) [2]. The quality registers provide a unique possibility to survey different aspects of health care and health care outcomes. In addition, quality registers can be used to conduct research, to improve quality of health care, and to manage health care services [7]. Clearly, it is important that data in the registers are valid and representative [8]. The major part of quality registers are financed by the Swedish government and the Swedish Association of Local Authorities and Regions, government entities that have deemed it a national priority that quality registers should cover at least 80% of the population [7].

In Sweden, management of national quality registers is regulated by Swedish legislation and the National Board of Health and Welfare [7]. Collection and management of patient data in quality registers are regulated by the Swedish Patient Data Law, which charges health providers the responsibility of informing patients on the existence of a specific health register, the purpose of the register, and the type of data that are reported to the register. The patients are informed that their participation in the health register is voluntary and that removal of data is automatically granted if the patient desires [9].

### The Swedish Maternal Health Care Register

The Swedish Maternal Health Care Register (MHCR) is a national quality register established in 1999. In 2007, a substantial revision was performed of its variables, Web application, and technical solutions. The revised version of MHCR was launched on January 1, 2010. The MHCR collects pregnancy, delivery, and postpartum data, including individual data on the pregnant women, foetuses, and infants. In 2010 and 2011, 81% and 85%, respectively, of the pregnant population were registered in the MHCR (personal communication). The main bulk of data registered in MHCR is related to pregnancy and delivery, but data on lifestyle, education, and socio-economic factors are also reported. In accordance with the Swedish Patient Data Law [9], all antenatal care centres (ANC) are charged with informing each pregnant woman on the existence of the MHCR, its purpose, its content, and the fact that providing data is voluntary.

Data in the MHCR are entered on two different occasions by attending ANC midwives. Entering data into the MHCR is performed using a Web-based application specifically created for this purpose. To protect the integrity of the data, each midwife is provided with an individual user identity and a secure login procedure. The first dataset is entered when a pregnant woman registers in ANC. This dataset mainly includes information about background characteristics, such as educational level, weight, height, and smoking habits. On the first visit, Body Mass Index (BMI) is calculated using a software program built into the MHCR.

According to national recommendations for health care during pregnancy and after delivery, all women should be offered a postpartum meeting with a midwife in the ANC four to 16 weeks after delivery [10]. The second data entry takes place soon after the postpartum visit and includes items related to pregnancy, delivery, and the postpartum period. If a woman does not attend the voluntary postpartum visit, the midwife enters the second set of data at around 16 weeks postpartum using information from the medical records. The items in this second data entry address the outcomes of pregnancy and delivery.

Most of the registered items entered in the MHCR are data obtained from medical records manually registered by a midwife. The MHCR database is administrated by the Uppsala Clinical Research Centre (UCR), which specifically supports the maintenance of national quality registers and assists researchers using these registers.

No previous study has evaluated the validity of data included in the MHCR. As national quality registers are used for quality improvement and management within regional and local health care services as well as for research, it is important that the quality of data in the registers is high.

### Aims

This study investigate the validity of data entered in the MHCR. The study has three specific aims: *i*) to explore degree of coverage of specified variables; *ii*) to investigate internal validity of data, including sensitivity and specificity of binary variables; and *iii*) to identify potential systematic errors.

## Methods

### Study design and study sample

This cross-sectional observational study compared data on pregnancy and delivery using medical records and the MHCR. The Regional Ethical Board at Umeå University (Umeå, Sweden) approved the national study (Dno 2012-44-31 M).

A power estimation was performed to determine the sample size; to obtain kappa values of 0.6 (considering the null value of kappa to be 0.4) and to achieve 90% power, a sample size of 540 was required if the prevalence was 0.1 (or 0.9) and 220 if the prevalence was 0.5. However, kappa is very sensitive to prevalence and as the categorical variables vary considerably with respect to prevalence, a sample of 900 medical records was judged to be a sufficient sample size to respond to the research questions under study. This study uses a national sample comprising nine Swedish hospitals, 100 medical records from each hospital. The hospitals were selected because they provided a variation in geographic and demographic characteristics. In Sweden, there were 109,752 deliveries in 2011. The data collection was performed at hospitals representing delivery units ranging from 1,298 to 10,363 births in 2011 [11] and covered the northern and southern regions of Sweden. To some extent, the selection of hospitals was influenced by convenience, as most of the authors of this study constituted a subset of the board of the MHCR and are affiliated with five of the selected hospitals included in the study. These circumstances provided a better opportunity to supply instructions and support to the local administrators who were extracting the data from the medical records.

Inclusion criteria for the study were medical records of women with data on pregnancy and delivery both in the medical records and in the MHCR. Exclusion criteria were data lacking in either of these two data sources.

### Medical records of pregnancy, delivery, and the postpartum period

The software program Obstetrix^®^ is widely used in Sweden and contains pregnancy, delivery, and postpartum data, accounting for approximately 90% of medical records on pregnant women in Sweden. Other software programs used in clinical practice are Partus^®^ and Cosmic Birth^®^. A few clinics still document medical data using pen and paper.

### Data collection procedures

Before the start of the study, the heads of all participating clinics provided verbal consent to participate. After the consent was secured from the heads of the clinics, local administrators, one administrator at each hospital, were contracted to supply the data registration. Most of the local administrators were medical secretaries, but in a few hospitals midwives or other staff were contracted.

In 2011, data on 85% of all pregnant women were included in the MHCR. Therefore, the personal identity numbers of 120 consecutively delivered women were extracted from the birth log at each clinic to ensure that 100 women were identified from each clinic with data both in the medical records and in the MHCR. From the nine clinics, we selected 100 women per clinic who gave birth from March 1^st,^ and whose data were in their medical records and in the MHCR. The smaller clinics required a longer time to collect these data (March 1^st^ to May 29^th^) and the larger clinics required a shorter time (March 1^st^ to March 9^th^). Extracted data from the medical records for the 900 women were transferred in encrypted form to the UCR. The UCR combined the extracted data in the medical records with the corresponding data in the MHCR. The goal was to collect data on 100 women from each hospital; i.e., we wanted to have data from 900 medical records. For seven hospitals, data on 100 women were incomplete. Despite repeated reminders by e-mail and by telephone, no further data were delivered, resulting in a final dataset of extracted data from 878 medical records.

### Study protocol

An Excel^®^-protocol was developed by the authors to register categorical and numeric variables extracted from the medical records and to secure that data were extracted in a similar manner at all hospitals. In general, registration of data from the medical records into the Excel^®^-protocol was done manually by the local administrator. However, in one hospital data were electronically collected from the medical records and imported into the Excel^®^-protocol. Then the content of each Excel^®^-protocol was encrypted and sent to the UCR.

Data from Excel^®^-protocols and data from the MHCR were merged by the UCR using the personal identity number for each woman. To ensure that individuals could not be identified, the merged dataset was delivered to the authors with each individual given a unique serial number.

### Presentation of included variables

All variables included in the MHCR and the selected variables for this study are presented in Table 1. Some variables available in the MHCR were excluded for the validity control, such as variables regarding the postpartum period and variables with no corresponding data in the medical records (e.g., the variables of self-reported health before, during, and after pregnancy).

**Table 1:** **Presentation of all variables registered at first and second data entry in the Sweedish Maternal Health Care Register (MHCR)**

First data entry	Second data entry	
Data collected at first visit in antenatal care (ANC)	Data collected at postpartum visit in antenatal care (ANC) 4 to 16 weeks after delivery	
**Variables**	**Variables**	**Variables**
**Date of first visit in ANC** ^**a**^	**Live born child**	Treatment of psychiatric disorder
Country of birth	Still birth/termination of pregnancy	Questioned about exposure to violence
**No. of previous deliveries**	Date of delivery estimated by ultrasound	**Oral glucose tolerance test (OGTT) performed**
**Maternal weight (kilograms)** ^**b**^	**Estimated date of delivery (ultrasound)**	**2-hour value of plasma glucose at OGTT (mmol/L)**
**Maternal height (centimetres)** ^**b**^	Estimated date of delivery (last menstruation)	Diagnosis of gestational diabetes mellitus (GDM)
Smoking three months prior to pregnancy	**Ultrasound examination at gestational age 16-21 week**	**Date of delivery**
No. of cigarettes/day three months prior to pregnancy	**Combined Ultrasound and Biochemical screening (CUB)**	Maternal age at delivery
**Smoking at first ANC visit**	**Second trimester serum screening**	**Mode of delivery**
No. of cigarettes/day at first ANC visit	**Chorionic villus sampling (CVS)**	**If caesarean section, elective or emergency section**
Use of snuff three months prior to pregnancy	**Amniocentesis (AC)**	**Singleton birth/multiple births**
**Use of snuff at first ANC visit**	**Number of antenatal visits until estimated date of delivery (determined by ultrasound)**	**Birth weight (grams)** ^**c, d**^
**Assessment of use of alcohol prior to pregnancy with screening instrument Alcohol Use Disorder Identification Test (AUDIT)**	Number of midwives surveying the pregnant woman in ANC	**Gender of infant** ^**d**^
**AUDIT-score**	Use of authorized interpreter	Documented suspicion of intrauterine growth retardation
Education level	**Smoking at 32 weeks of gestation**	Postpartum visit at ANC
Employment status	No. of cigarettes/day at 32 gestational weeks	Date of postpartum visit at ANC
Self-rated health prior to pregnancy	**Use of snuff at 32 weeks of gestation**	Maternal body weight at postpartum visit at ANC (kilograms)
	**Maternal weight (in kilograms), last data entry after 35 gestational weeks**	Self-rated health during pregnancy
	Participated in prenatal education group (pregnant woman)	Self-rated health postpartum
	Participated in prenatal education group (partner)	Breast feeding at 4 weeks postpartum
	Counselling due to fear of childbirth	

Variables presented in bold text were selected for the comparison of data in medical records and in MHCR.

^a^Gestational age at registration in ANC is calculated by the software program.

^b^Body Mass Index (BMI) at registration in ANC is calculated by the software program.

^c^Foetal growth proportionality – i.e., appropriate for gestational age (AGA), large for gestational age (LGA), and small for gestational age (SGA) – is calculated by the software program.

^d^In cases of multiple births, birth weight and gender are also registered for second twin.

Most of the categorical variables in the MHCR had the response options of “yes”, “no”, or “don’t know”. However, two variables had other response options: “mode of delivery” (“caesarean section”, “instrumental vaginal delivery”, or “non-instrumental vaginal delivery”) and the variable “gender” (“girl”, “boy”, or “unknown gender”). Three of the categorical variables with response options “yes”, “no”, or “don’t know” had an additional question if the response “yes” was noted. These variables had the following additional options: *i*) Alcohol Use Disorder Identification Test (AUDIT) scores; *ii)* the options elective caesarean section (CS) or emergency CS, if mode of delivery was registered as CS; and *iii)* the two-hour plasma glucose value was requested if an oral glucose tolerance test (OGTT) had been performed.

Quantitative variables were registered as continuous numeric values. Birth weight was registered in grams. Maternal body weight was recorded in whole kilograms and maternal height in centimetres. AUDIT-scores ranged from 0 to 40. Variables addressing dates were registered in a pre-set calendar format. Some deliveries were multiple births. Data on first twin, such as mean birth weight and mode of delivery, were included in the presentation of singleton pregnancies. Mean birth weight for second twin was also calculated.

### Control of data registered in the protocol

To investigate to what extent data from the medical records had been correctly registered in the Excel^®^-protocol, a second data extraction was performed (i.e., re-collection of data). Three of the participating hospitals – Östersund Hospital (Östersund), Södersjukhuset (Stockholm), and Umeå University Hospital (Umeå) – were selected for this control procedure. Two of the authors (KP and IH, both midwives with extensive experience with ANC) performed this re-collection of data. An identical Excel^®^-protocol as used for the first data collection from medical records was used for this second data collection procedure. The goal was to include every second woman from the primary dataset from each of the three selected hospitals in this second validation procedure of data (i.e., data were collected from medical records on 50 women from each hospital, resulting in data from 150 medical records).

### Statistical analysis

Data from the medical records were considered the gold standard. The proportions of available data in the medical records and in the MHCR and the proportions of data available in both data sources were calculated for each variable. In addition, the proportion of cases where the medical records and the MHCR presented identical information was calculated for each variable. For the subset of data (re-collected dataset) where the categorical variables with a subsequent explorative question in the case of a “yes” response, the number of “yes” responses constituted the denominator in the calculations. Degree of agreement was estimated using Cohen’s kappa for categorical data and Pearson’s correlation coefficient was used for normally distributed, continuous data. Spearman’s correlation coefficient was used to evaluate dates. Sensitivity and specificity were analysed for binary variables. Sensitivity was defined as the proportion of actual positives, that were correctly identified as such. Specificity was defined as the proportion of negatives that were correctly identified as such. Sensitivity and specificity were analysed for binary variables. SPSS version 19 was used for all calculations. The level of significance was set at 0.05.

## Results

### Background presentation

Corresponding data on pregnancy and delivery from medical records and the MHCR were collected from 878 medical records at nine hospitals. These hospitals and their characteristics are presented in Table 2. The number of deliveries at the included hospitals corresponds to 28.0% of the total number of deliveries in Sweden in 2011. The data collected from medical records included mean age (30.7 years, SD ±5.0), mean BMI (24.6, SD ±4.6), and mean birthweight of infant (3515 g, SD ± 568). Eleven pregnancies were multiple births. The mean gestational age was 278.2 days (SD ±12.5) or 39.7 weeks (SD ±1.8) for singleton births and 241.6 days (SD ±36.2) or 34.5 weeks (SD ±5.2) for multiple births. Mean birth weight of second twin was 1810 g (SD ±1003).

**Table 2:** **Characteristics of the nine participating hospitals and number of medical records extracted at each hospital**

City	Participating hospital	Level of health care	Inhabitants/km ^2^2011 ^a^	No. of births 2011 ^b^(%) ^c^	No. of medical records (%) ^d^
Göteborg	Sahlgrenska University Hospital	University	66.8	10363 (9.4)	91 (10.4)
Halmstad	Halmstad Hospital	County	55.6	1799 (1.6)	96 (10.9)
Jönköping	Ryhov Hospital	County	32.4	2075 (1.9)	99 (11.3)
Stockholm	Karolinska University Hospital	University	320.5	4642 (4.2)	96 (10.9)
Stockholm	Södersjukhuset	University	320.5	7331 (6.7)	98 (11.2)
Sundsvall	Sundsvall Hospital	Regional	11.2	1536 (1.4)	100 (11.4)
Umeå	Umeå University Hospital	University	4.7	1817 (1.6)	100 (11.4)
Örebro	Örebro University Hospital	University	33.1	2867 (2.6)	99 (11.3)
Östersund	Östersund Hospital	Regional	2.6	1298 (1.2)	99 (11.3)
				30728 (28.0)	878 (100%)

^a^Population density in catchment area. Data from “Inhabitants per kilometer^2^” [Internet] Statistics Sweden; 2011 (cited 2013, June 6) http://www.scb.se/Pages/SSD/SSD_SelectVariables340487.aspx?px_tableid = ssd_extern%3aBefArealTathetKon&rxid = ca8cabdd-0d60-488b-b047-4b5c5a89dcb5.

^b^Data from National Board of Health and Welfare’Graviditeter, förlossningar och nyfödda barn. Medicinska Födelseregistret 1973-2011. Assisterad befruktning 1991 – 2010’ [in Swedish] http://www.socialstyrelsen.se/publikationer2013/2013-3-27.

^c^Proportions are calculated by using the total no of births in Sweden 2011 (N = 109 752) as denominator.

^d^Proportions are calculated by using the total no of medical records as denominator.

### Degree of coverage of data in medical records and in the MHCR

The degree of coverage of all investigated variables is presented in Table 3. The degree of coverage of variables included in medical records varied from 48% to 100% and most variables presented high degree of coverage in medical records. There was a high degree of coverage for the categorical variable OGTT (98.9%) in medical records. However, there was a lower degree of coverage for the associated variable “OGTT two-hour value of plasma glucose” (48.0%) in medical records.

**Table 3:** **Data in medical records and the Sweedish Maternal Health Care Register (MHCR); comparison between the two data-sets using correlation analysis, and analysis of sensitivity and specificity for binary variables**

Variable	Data source: Medical records		Data source: MHCR		Data available in both data sources		Identical information in both data sources		Correlation ^a^	Sensitivity	Specificity
	n	%	n	%	n	%	n	%			
***Variables collected at first antenatal care (ANC) visit***											
Date of first visit in ANC (numerical)	877	99.9	868	98.9	867	98.7	685	79.0	0.878 (S)		
No of previous deliveries (numerical)	878	100	867	98.7	867	98.7	840	96.8	0.971 (P)		
Maternal weight at first ANC visit (numerical)	862	98.1	855	97.4	847	96.4	798	94.2	0.990 (P)		
Maternal height (numerical)	872	99.3	862	98.2	860	97.9	834	97.0	0.982 (P)		
Smoking at first ANC visit (Yes/No)	875	99.7	872	99.2	868	98.9	843	97.1	0.742 (C)	0.650	0.995
Use of Snuff at first ANC visit (Yes/No)	878	100	871	99.2	871	99.2	861	98.9	0.540 (C)	0.429	0.998
Assessment of alcohol screening prior to pregnancy (AUDIT) (Yes/No)	802	91.3	859	97.8	788	89.7	691	87.7	0.480 (C)	0.986	0.393
If Yes, AUDIT score (numerical)^b^	650/643	98.9	777/771	99.2	621	95.5	600	96.6	0.989 (P)		
***Variables collected at 4 to 16 weeks postpartum***											
Prenatal diagnostics											
Amniocentesis (AC) (Yes/No)	875	99.7	791	90.1	788	89.7	772	98.0	0.754 (C)	0.743	0.991
Chorion Villus Sampling (CVS) (Yes/No)	875	99.7	790	90.0	787	89.6	778	98.9	0.176 (C)	0.167	0.995
Combined Ultrasound and Biochemical screening (CUB) (Yes/No)	780	88.8	791	90.1	700	89.7	665	95.1	0.888 (C)	0.936	0.957
Second trimester Serum Screening (Yes/No)	849	96.7	790	90.0	767	87.4	671	87.4	0.002 (C)	0.043	0.958
Ultrasound examination at 16 – 21 gestational weeks (Yes/No)	862	98.2	791	90.1	779	88.6	755	96.9	0.064 (C)	0.979	0.800
Estimated date of delivery (ultrasound) (numerical)^c^	871	99.2	874	99.5	868	98.9	781	90.0	0.946 (S)		
Oral Glucose Tolerance Test (OGTT) performed (Yes/No)	869	98.9	877	99.9	868	98.9	842	97.0	0.854 (C)	0.880	0.982
If Yes, 2-hour value of plasma glucose at OGTT (numerical)^d^	100/48	48.0	104/97	93.3	46	46.0	34	73.9	0.902 (P)		
Smoking at 32 gestational weeks (Yes/No)	858	97.7	876	99.8	856	97.5	849	99.1	0.864 (C)	0.821	0.998
Use of Snuff at 32 gestational weeks (Yes/No)	832	94.8	876	99.8	830	94.5	826	99.5	0.712 (C)	0.625	0.999
Maternal weight, last data entry after 35 gestational weeks (numerical)	777	88.5	843	96.0	760	86.6	706	92.9	0.989 (P)		
No. of ANC visits until estimated date of delivery (numerical)	877	99.9	868	98.9	867	98.7	627	72.3	0.915 (P)		
Date of delivery (numerical)	878	100	878	100	878	100	842	95.9	0.989 (S)		
Live born child (Yes/No)	878	100	878	100	878	100	874	99,5	0.598 (C)	0.999	0.500
Birth weight (numerical)	876	99.8	869	99.0	868	98.9	813	93.7	0.989 (P)		
Gender of infant (Boy/Girl/Sex unknown)	878	100	874	99.5	874	99.5	862	99.2	0.973 (C)		
Singleton birth/multiple births	877	99.9	878	100	877	99.8	875	99.7	0.908 (C)		
Mode of delivery (vaginal/instrumental vaginal/caesarean section)	876	99.8	876	99.8	874	99.5	857	98.0	0.946 (C)		
If caesarean section, elective CS/emergency CS^e^	130/115	88.5	129/128	99.2	110	84.6	102	92.7	0.841 (C)		

Comparison between the two data-sets using correlation analysis, and analysis of sensitivity and specificity for binary variables.

^a^Correlation analysis: C = Cohen’s kappa, P = Pearson´s correlation coefficient, S = Spearman`s correlation coefficient;

^b^Measures are calculated for those who have undergone alcohol screening (n = 650). The denominator is the total no of “Yes” responses. Denominator in the Medical records =650. Denominator in the MHCR = 771.

^c^Measures are calculated for those who have undergone ultrasound.

^d^Measures are calculated for those who have undergone OGTT. The denominator is the total no of “Yes” responses. The denominator for the medical records = 100. The denominator for the MHCR = 104.

^e^Measures are calculated for those who have undergone caesarean section. The denominator is the total no of “Yes” responses. The denominator for the medical records = 130. The denominator for the MHCR = 129.

Degree of coverage of data registered in the MHCR varied between 90.0% and 100%. The variables with a relatively lower degree of coverage in the MHCR, although in fact a high degree of coverage, addressed various forms of prenatal diagnostics with a degree of coverage of approximately 90%.

Data available in both data sources (medical records and MHCR) ranged from 46.0% to 100%. Variables with complete data in both data sources were variables addressing date of birth and whether the child was born alive or stillborn. Other variables with a high level of data available in both data sources included “singleton birth/multiple births” (99.8%), “mode of delivery” (99.5%), and “gender of child” (99.5%).

### Agreement of data in medical records and in the MHCR

Identical data in both data sources ranged from 73.9% to 99.7%. For more than half of the investigated variables (17 of 27 variables), agreement of data in both data sources reached 95% or more. Five variables reached an agreement of data in both data sources of less than 90% (Table 3). Variables with the highest frequencies of identical information in the MHCR and in the medical records were mainly data related to delivery, such as “singleton birth/multiple births”, “live born child”, and “gender of child”. For the eleven multiple births, the agreement of birth weights of second twin was identical in both data sources (100%).

Table 4 presents the comparison between the primary data collection from the medical records and the re-collection of variables from 150 reinvestigated medical records. The degree of coverage of data in the reinvestigated medical records ranged from 86.7% to 100%; frequencies of available data in medical records were similar or improved at the re-collection with one exception. The re-collection contributed to an improvement of the number of variables with 100% available data in both data sources. In addition, the number of variables with identical data increased in comparison to the first data collection. Identical data in both data sources ranged from 64.0% to 100%. Twenty-two of the 27 variables reached agreement between data sources for 95.0% or more in the reinvestigated data collection. Furthermore, the re-collection of data improved the agreement of data, resulting in only two of the 27 variables showing an agreement in both data sources to less than 90% in the reinvestigated material.

**Table 4:** **Comparison between primary collection and re-collection of data from medical records using correlation analysis, and analysis of sensitivity and specificity for binary variables**

Variable	Medical records ^a^		Medical records re-collection ^b^		Data available in both data sources		Identical information in both data sources		Correlation ^c^	Sensitivity	Specificity
	n	%	n	%	n	%	n	%			
***Variables collected at first antenatal care (ANC) visit***											
Date of first visit in ANC (numerical)	150	100	150	100	150	100	116	77.3	0.773 (S)		
No of previous deliveries (numerical)	150	100	150	100	150	100	149	99.3	0.988 (P)		
Maternal weight at first ANC visit (numerical)	147	98.0	148	98.7	147	99.3	146	99.3	0.995 (P)		
Maternal height (numerical)	149	99.3	149	99.3	149	100	148	99.3	1.000 (P)		
Smoking at first ANC visit (Yes/No)	149	99.3	149	99.3	149	100	148	99.3	0.794 (C)	^**d**^	1.000
Use of Snuff at first ANC visit (Yes/No)	150	100	150	100	150	100	146	97.3	0.793 (C)	0.667	1.000
Assessment of alcohol screening prior to pregnancy (AUDIT) (Yes/No)	130	86.7	130	86.7	130	86.7	121	93.1	0.729 (C)	0.972	0.136
If Yes, AUDIT score (numerical)^e^	113/109	96.5	108/106	98.1	102	90.3	100	98.0	0.987 (P)		
***Variables collected at 4 to 16 weeks postpartum***											
Prenatal diagnostics											
Amniocentesis (AC) (Yes/No)	150	100	150	100	150	100	150	100	1.000 (C)	1.000	0.983
Chorion Villus Sampling (CVS) (Yes/No)	150	100	150	100	150	100	150	100	1.000 (C)	^**d**^	0.992
Combined Ultrasound and Biochemical screening (CUB) (Yes/No)	147	98.0	149	99.3	147	98.6	142	96.6	0.912 (C)	0.919	0.941
Second trimester Serum Screening (Yes/No)	148	98.7	150	100	148	98.7	148	100	^f^		
Ultrasound examination at 16 – 21 gestational weeks (Yes/No)	147	98.0	147	98.0	147	98.0	145	99.0	0.246 (C)	0.975	^**d**^
Estimated date of delivery (ultrasound) (numerical)^g^	147	100	147	100	147	100	145	98.7	0.955 (S)		
Oral Glucose Tolerance Test (OGTT) performed (Yes/No)	149	99.3	149	99.3	148	98.0	144	98.0	0.819 (C)	1.000	0.986
If Yes, 2-hour value of plasma glucose at OGTT (numerical)^h^	13/10	77.0	10/9	90.0	9	69.2	9	100	1.000 (P)		
Smoking at 32 gestational weeks (Yes/No)	145	96.7	145	96.7	145	100	145	100	1.000 (C)	1.000	1.000
Use of Snuff at 32 gestational weeks (Yes/No)	145	96.7	145	96.7	145	100	144	99.3	0.797 (C)	1.000	1.000
Maternal weight, last data entry after 35 gestational weeks (numerical)	142	94.7	141	94.0	141	99.3	137	97.2	1.000 (P)		
No. of ANC visits until estimated date of delivery (numerical)	150	100	150	100	150	100	96	64.0	0.890 (P)		
Date of delivery (numerical)	150	100	150	100	150	100	149	99.3	0.975 (S)		
Live born child (Yes/No)	150	100	150	100	150	100	150	100	^f^	1.000	1.000
Birth weight (numerical)	150	100	150	100	150	100	140	93.3	0.997 (P)		
Gender of infant (Boy/Girl/Sex unknown)	150	100	150	100	150	100	149	99.3	0.987 (C)		
Singleton birth/multiple births	150	100	150	100	150	100	150	100	1.000 (C)		
Mode of delivery (vaginal/instrumental vaginal/caesarean section)	150	100	150	100	150	100	149	99.3	0.983 (C)		
If caesarean section, elective CS/emergency CS^i^	23/22	95.7	23/23	100	22	95.7	22	100	1.000 (C)		

^a^Primary collection of data from medical records.

^b^Re-collection of data from medical records.

^c^Correlation analysis: C = Cohen’s kappa, P = Pearson´s correlation coefficient, S = Spearman`s correlation coefficient.

^d^Sensitivity or specificity not possible to calculate since one or more of the cells in the calculation includes zero.

^e^Measures are calculated for those who have undergone alcohol screening. The denominator is the total no of “Yes” responses. Denominator in the Medical records (n = 113), denominator in the MHCR (n = 108).

^f^Cohen’s kappa is not calculated as one of the variables is a constant.

^g^Measures are calculated for those who have undergone ultrasound.

^h^Measures are calculated for those who have undergone OGTT. The denominator is the total no of “Yes” responses. Denominator for the medical records (n = 13), denominator for the MHCR (n = 10).

^i^Measures are calculated for those who have undergone caesarean section. The denominator is the total no of “Yes” responses. Denominator for the medical records (n = 23), denominator for the MHCR (n = 23).

### Sensitivity and specificity

Analyses of sensitivity and specificity were performed on binary variables (Table 3). The medical record was considered to represent the true value. Sensitivity varied from 0.043 (second trimester screening) to 0.999 (live born child), and specificity ranged from 0.393 (assessment of alcohol screening prior to pregnancy) to 0.999 (use of snuff at 32 gestational weeks). For nine out of the 12 binary variables, specificity was 0.900 or higher, whereas only four out of 12 binary variables had a sensitivity of 0.900 or higher.

### Systematic errors

Possible systematic errors were identified for two variables: “second trimester serum screening” and “number of ANC visits”. The variable “second trimester serum screening” demonstrated identical information in both data sources for 87.4%. One of the hospitals reported an unexpected large number of performed second trimester screenings in both data sources. The reported number of “second trimester serum screening” was not consistent with the clinical practice, so we discussed this issue with the midwives working in the catchment area of this hospital. These discussions revealed that that the variable “second trimester serum screening” probably had been misunderstood, resulting in incorrect reporting of data.

The variable “number of ANC visits” showed an agreement of data in both data sources for 72.3% of the cases. The information addressing this variable in the Web application was defined as the number of visits to see a midwife at an ANC (noted on the ANC registration) until estimated date of delivery as established by ultrasound (not the actual date of birth). As pregnant women may meet other health care providers during pregnancy, such visits may have been included in the figure entered in the MHCR. A misfit of ± 1 visit was seen in 19.3% of the cases. The variation of misfiting values ranged from -7 visits to + 8 visits.

## Discussion

This is the first time that the validity of data entered in the MHCR has been investigated. Data from 878 medical records were compared with corresponding data registered in the MHCR. The information registered in the medical records was regarded as the gold standard. Data entered in the MHCR presented a strong correlation to corresponding data in the medical records. More than half of the variables under study demonstrated identical information in both data sources to a level of 95% or more. Five of the 27 studied variables showed an agreement of less than 90% in both data sources. A second re-collection of the same variables of a subset of 150 medical records of the original sample, performed to further validate the primary data collection in this study, increased the number of variables with identical information in both data sources. Possible sources of systematic errors in the MHCR were identified for two variables.

### Degree of coverage of data

The findings of this study presented a sufficient degree of coverage of data in the medical records under study. Data from the medical records have been transferred to the Swedish Medical Birth Register (MBR) since 1973. Previous studies have shown that most variables in the MBR demonstrate sufficient degree of coverage of data [12, 13].

The estimated proportion of registered pregnancies in MHCR during 2010 and 2011 were 81% and 85%, respectively (personal communication). Missing MHCR data could be the result of midwives failing to enter data for all pregnant women as this work task is not compulsory and the fact that providing data is voluntarily (i.e., pregnant women can choose to opt out). However, missing data related to opting out is considered a minor issue (personal communication).

The degree of coverage of data entered in the MHCR was high for most variables in our study. The data in the MHCR were entered by the midwife working in the ANC; some information was available in the medical records and some information was provided orally by the pregnant woman. The variables regarding prenatal diagnostics in the MHCR demonstrated a relatively lower degree of coverage than other included variables, although it was still high. A possible explanation for this relatively lower degree of coverage may be the design of this question in the MHCR Web application. Only after the midwife registered “yes” for the question “Have any foetal diagnostics been performed?” is the second option displayed. In the Swedish MBR, an improvement of data quality regarding amniocentesis and chorionic villus sampling was found when the location of these variables in the medical records was changed [11]. Hence, rephrasing and redesigning these questions in the Web application may further improve the degree of coverage of data for variables related to prenatal diagnostics.

To our knowledge, no previous studies have monitored how primary data are registered in the medical records or have investigated the validity of primary data in relation to data included in the medical records. Our study shows that some variables demonstrated a higher degree of coverage in the MHCR than in the medical records. Some studies that use vital statistics databases for perinatal epidemiology have a major limitation: the data these studies use, although considered the gold standard, have not been evaluated for their reliability and validity [14].

### Agreement between data sources

The agreement of data in both data sources was high for most variables (Tables 3 and 4). To analyse correlation of categorical data, Cohen’s kappa was used. Cohen’s kappa is defined only for a square table and is strongly influenced by prevalence (e.g., number of “yes” responses). When there is a high level of correlation between two variables and when one of four cells is empty, the performance of Cohen’s kappa declines. This decline was the case for the variable “use of snuff”, where Cohen’s kappa was calculated to 0.540, although data were identical for 98.9% of cases in the medical records and in the MHCR. Another example was the variable “chorionic villus sampling”, where Cohen’s kappa was calculated to be 0.176, although the proportion of identical data in medical records and in the MHCR reached as high as 98.9%. In these cases, the proportion of identical information in both data sources provided more valuable information than Cohen’s kappa provided.

Our findings of agreement between the data sources were similar to the findings reported in a pilot study that assessed data quality in the Uniform Data Set (UDS) used by the American Association of Birth Centers [15]. In this pilot study, a care provider entered data online on four occasions; the data addressed demographic characteristics, risk factors, process of care, and maternal and infant outcomes. The agreement of variables from medical records and the UDS varies from 87.5% to 100%.

In an American evaluation of the use of electronic health records in emergency medical services, electronic data processing was compared to manual data processing. The results show good to excellent agreement between the two approaches [16]. In the Swedish setting, there is a disadvantage when data are entered in the MHCR, as data from the medical records currently cannot be automatically exported to the MHCR. All registrations in the MHCR are made manually by midwives in an ANC. Despite these potential sources of manual mistakes when registering data, the findings in our study indicate that the accuracy of data registered in the MHCR reaches a level of good to very good.

### Sensitivity and Specificity

Variables characterized by one of the binary response options (“yes” and “no”) demonstrated either a high specificity and a low sensitivity or a low specificity and a high sensitivity. Binary variables demonstrating a high specificity and a low sensitivity were “use of snuff”, “smoking”, “chorion villus sampling”, and “second trimester screening”. In contrast, variables characterized by a majority of “yes” responses demonstrated high sensitivity and low specificity (i.e., “assessment of alcohol screening prior to pregnancy”, “ultrasound examination at 16-21 gestational weeks”, and “live born child”). These results indicate that midwives performing data entry are more prone to enter results that are expected than unexpected. Similarly, an American study investigating the correctness of data in a computerized perinatal database found that there is greater likelihood to overlook a given diagnosis than to score positive a disease that does not occur [17]. A review on quality of data in perinatal health databases, including 43 validation studies, shows that most conditions and procedures demonstrate high specificities, indicating few false positives [18]. Most of the binary variables in our study demonstrated a low prevalence of the investigated outcome. This finding may explain why only four of 12 variables showed a sensitivity exceeding 0.900.

### Systematic errors

This study revealed two potential systematic errors when registering data in the MHCR. First, the analysis demonstrated a misinterpretation at one of the participating hospitals regarding the registration of “second trimester serum screening” in the catchment area. An English study reveals that some midwives (7.7%) believe that they are not sufficiently prepared to inform patients about available foetal screening methods. The majority of midwives feel they are prepared to offer their patients information about screening, but when testing the level of knowledge of the conditions detectable by the available screening tests, the knowledge does not match the preparedness [19]. The situation presented in the English study might be applicable to the Swedish setting as well. The available methods for prenatal screening and prenatal diagnostics have rapidly increased over the last decade, resulting in more complex information and counselling needs [10], so some midwives working in an ANC might not have had sufficient knowledge to correctly enter data in the MHCR. The second possible systematic error found was when addressing the number of ANC visits during pregnancy. A quality study of the Swedish Medical Birth Register found that information on the number of ANC visits is missing in approximately 11% of the cases [10]. Our study found that the degree of coverage of this variable was high for both data sources, but the agreement between the data sources was not as high. A possible source for the lower accuracy could be related to insufficient instructions in the MHCR manual. Most of the incorrect values ranged ± one visit; a possible explanation for this is that visits after 40 gestational weeks or visits to the outpatient specialised clinic might have been included in the MHCR data. Improvements in the MHCR user manual might increase the level of correct data in the MHCR.

### Clinical importance

Quality register data are used for quality improvement and management within the health services as well as for research purposes. Therefore, it is of considerable importance that the improvements, decision-making, and results presented must be grounded in reliable and valid data. The benefit of the MHCR is the composition of the data, which include demographic, medical, and psychological aspects of the pregnancy, the delivery, and the postpartum period. Additionally, the data may be presented on a local, regional, and national level of the ANC, enabling comparisons of provided health care and outcomes of pregnancy and delivery. Despite manually registering data in the MHCR, the vast majority of variables included in the MHCR show very good agreement with corresponding information in the medical records. The findings in this study indicated that the data available from the MHCR are reliable enough to be used in clinical quality work and for research purposes.

### Further studies

As the data are registered manually in the MHCR by midwives in an ANC, the experiences of midwives is important to address – How do midwives experience this work? Furthermore, it would be of interest to find out how data available in the MHCR are used for clinical improvements and quality aspects of health care at the local and regional levels of the ANC.

### Methodological considerations

One of this study’s strengths is its design. Data were extracted and analysed in two steps: a primary data extraction from 878 medical records and a secondary data extraction of the same variables for a subset of 150 medical records from the primary sample. The re-collection of data was performed by two midwives (i.e., two of this paper’s authors) with extensive experience working in an ANC. This experience may have contributed to the improved quality of the data with increased statistical agreement between datasets. Data extracted by professionals other than midwives might be less accurate as these professionals may have much less experience evaluating and registering this type of data, a disadvantage that may have led to problems identifying the correct information.

Another strength of this study is the geographical variation of the included hospitals. The data extraction was performed at clinics in large cities as well as in small clinics located in more rural areas in Sweden. The selection of hospitals, in part, was determined by convenience as some of the authors were affiliated with five of these hospitals. Four other hospitals were selected with complementary characteristics in relation to the first five selected hospitals. The first author had close contact with the administrators at these hospitals in order to enhance the quality of the data collection. We believe that the selected hospitals sufficiently reflect the general characteristics of clinical settings in contemporary hospitals and ANC in Sweden.

The goal was to collect data for 900 medical records, 100 medical records from each hospital. In 2011, the degree of coverage of data was 85% in the MHCR (personal communication); that is, data were not available in the MHCR for 15% of pregnant women in Sweden for 2011. To identify 100 consecutive individuals with data in both medical records and the MHCR, we first collected the personal identity number of 120 individuals in the birth logs (from March 1), resulting in the identification of 100 women who had delivered at each hospital. Despite considerable efforts, this goal was not achieved as some administrators did not fully complete the Excel-protocols. Administrators of seven of the nine hospitals did not provide complete datasets. However, the number of missing cases (n = 22) corresponds to 2.4% of the goal, indicating that these missing cases could not have critically influenced the results of this study. Mean background characteristics on maternal age, height, weight, and BMI were 30.7 yrs, 166.2 cm, 67.9 kg, and 24.6 kg/m^2^, respectively in our study. The corresponding results in the MHCR for 2011 (N = 89 313) were 30.7 yrs, 166.2 cm, 68.4 kg, and 24.7 kg/m^2^, indicating that the study sample was representative for the year under study (personal communication).

## Conclusions

Comparing data from medical records – the gold standard – with data registered in the MHCR, we found that most variables in the MHCR demonstrated good to very good degree of coverage of data, agreement, and internal validity. Hence, data from the MHCR may be regarded as reliable when used for evaluation, planning, and decision-making in Swedish maternal health care services as well as for research purposes.
